# Fibroblast growth factor-2, but not the adipose tissue-derived stromal cells secretome, inhibits TGF-β1-induced differentiation of human cardiac fibroblasts into myofibroblasts

**DOI:** 10.1038/s41598-018-34747-3

**Published:** 2018-11-09

**Authors:** Tácia Tavares Aquinas Liguori, Gabriel Romero Liguori, Luiz Felipe Pinho Moreira, Martin Conrad Harmsen

**Affiliations:** 1 Laboratório de Cirurgia Cardiovascular e Fisiopatologia da Circulação (LIM-11), Instituto do Coração (InCor), Hospital das Clinicas HCFMUSP, Faculdade de Medicina, Universidade de Sao Paulo, Sao Paulo, SP Brazil; 2 University of Groningen, University Medical Center Groningen, Department of Pathology and Medical Biology, Groningen, The Netherlands

## Abstract

Transforming growth factor-β1 (TGF-β1) is a potent inducer of fibroblast to myofibroblast differentiation and contributes to the pro-fibrotic microenvironment during cardiac remodeling. Fibroblast growth factor-2 (FGF-2) is a growth factor secreted by adipose tissue-derived stromal cells (ASC) which can antagonize TGF-β1 signaling. We hypothesized that TGF-β1-induced cardiac fibroblast to myofibroblast differentiation is abrogated by FGF-2 and ASC conditioned medium (ASC-CMed). Our experiments demonstrated that TGF-β1 treatment-induced cardiac fibroblast differentiation into myofibroblasts, as evidenced by the formation of contractile stress fibers rich in αSMA. FGF-2 blocked the differentiation, as evidenced by the reduction in gene (*TAGLN*, p < 0.0001; *ACTA2*, p = 0.0056) and protein (αSMA, p = 0.0338) expression of mesenchymal markers and extracellular matrix components gene expression (*COL1A1*, p < 0.0001; *COL3A1*, p = 0.0029). ASC-CMed did not block myofibroblast differentiation. The treatment with FGF-2 increased matrix metalloproteinases gene expression (*MMP1*, p < 0.0001; *MMP14*, p = 0.0027) and decreased the expression of tissue inhibitor of metalloproteinase gene *TIMP2* (p = 0.0023). ASC-CMed did not influence these genes. The proliferation of TGF-β1-induced human cardiac fibroblasts was restored by both FGF-2 (p = 0.0002) and ASC-CMed (p = 0.0121). The present study supports the anti-fibrotic effects of FGF-2 through the blockage of cardiac fibroblast differentiation into myofibroblasts. ASC-CMed, however, did not replicate the anti-fibrotic effects of FGF-2 *in vitro*.

## Introduction

Fibroblasts are the most abundant cell type in the heart and regulate the homeostasis of the extracellular matrix (ECM). The ECM provides the architecture of cardiac tissue, it supports the structural integrity and it regulates cellular communication and function. A major component of the ECM is collagen which is deposited primarily by cardiac fibroblasts^1^. Pathological stimuli, such as myocardial infarction, disrupt the cardiac tissue homeostasis^2^ which predisposes the onset and progression of fibrosis^3,4^ by the activation and differentiation of fibroblasts into myofibroblasts^1,5^. Cardiac fibrosis features the production and deposition of excessive amounts of extracellular matrix by cardiac myofibroblasts. Fibroblast activation and subsequent differentiation into myofibroblasts are primarily driven by transforming growth factor-β (TGF-β) and contributes to the pro-fibrotic cardiac microenvironment^6–10^.

To date, no therapies exist that prevent or reverse fibrosis *in vivo*, yet it is possible to antagonize TGF-β signaling with specific growth factors such as FGF-2. Fibroblast growth factor 2 (FGF-2) is relevant in wound healing processes *in vivo*^11–13^ and *in vitro*^14–17^ to stimulate proliferation of tissue cells, connective tissue fibroblasts and promote angiogenesis, while it suppresses apoptosis. Moreover, FGF-2 antagonizes TGF-β signaling and thus affects fibrosis, albeit in early stages^18^. In fibroblasts of various origins, FGF-2 suppressed the expression of *TGFB1*^19^ and its protein^16,20^, that also suppressed the deposition of collagen^21,22^. All these studies suggest that FGF-2 is an attractive molecule to target in TGF-β regulated fibrotic disease.

Mesenchymal stromal cells (MSC), such as adipose tissue-derived stromal cells (ASC), as well as their conditioned medium, have been shown to improve cardiac remodeling and thus to modulate cardiac fibrosis^23–30^. Because ASC release a series of anti-fibrotic factors, among which are FGF, IGF and HGF^31–34^, a possible mechanism underlying their anti-fibrotic effect could be to antagonize TGF-β signaling and thus the inhibition of the transformation of fibroblasts into myofibroblasts as well as the reduction of extracellular matrix production.

In a previous study, we demonstrated that TGF-β1-induced differentiation of dermal fibroblasts to myofibroblasts could be modulated by adipose tissue-derived stromal cells’ conditioned medium (ASC-CMed)^35^. Therefore, we hypothesized that cardiac fibroblast differentiation into myofibroblast could also be abrogated by ASC-CMed.

## Results

### Formation of F-actin stress fibers in differentiating NHCF-V is refractory to suppression by ASC-CMed

**Figure 1:**
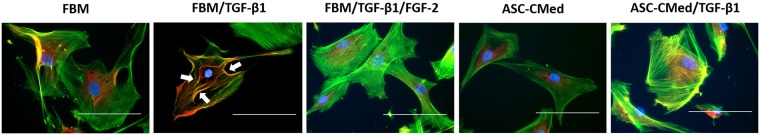
Formation of F-actin stress fibers in differentiating NHCF-V is refractory to suppression by ASC-CMed. Representative immunofluorescence micrographs of human cardiac fibroblasts (NHCF-V) under stimulation with TGF-β1 or co-stimulation with TGF-β1 and FGF-2, both in FBM and ASC-CMed, for 5 days. Upon TGF-β1 stimulation, cells developed transcellular αSMA-expressing stress fibers (arrows). ASC-CMed did not inhibit the development of stress fibers. Blue: DAPI; Green: phalloidin; Red: αSMA. Scale reference: 100 μm.

After five days of pro-fibrotic stimulus, NHCF-V undergo myofibroblast differentiation, phalloidin detected F-actin and cells had an increase in αSMA expression (Fig. 1). Human cardiac fibroblast without TGF-β1 stimuli had a mild phalloidin staining distributed all over the cytoplasm, on the other hand, the TGF-β1 stimulated cells had the detection of phalloidin in the contractile fibers which had colocalization with the αSMA staining. FGF-2, but not ASC-CMed, could inhibit the phalloidin and αSMA in the contractile fibers. The expression of αSMA seems to be more evident in the TGF-β1 stimulated cells. As expected, TGF-β1 induced myofibroblast differentiation of cardiac fibroblasts which leads to the formation of smooth actin fibers at cytoskeletal level. Only FGF-2 inhibited the formation of these fibers.

### FGF-2, but not ASC-CMed, inhibits the expression of mesenchymal markers in NHCF-V stimulated with TGF-β1

**Figure 2:**
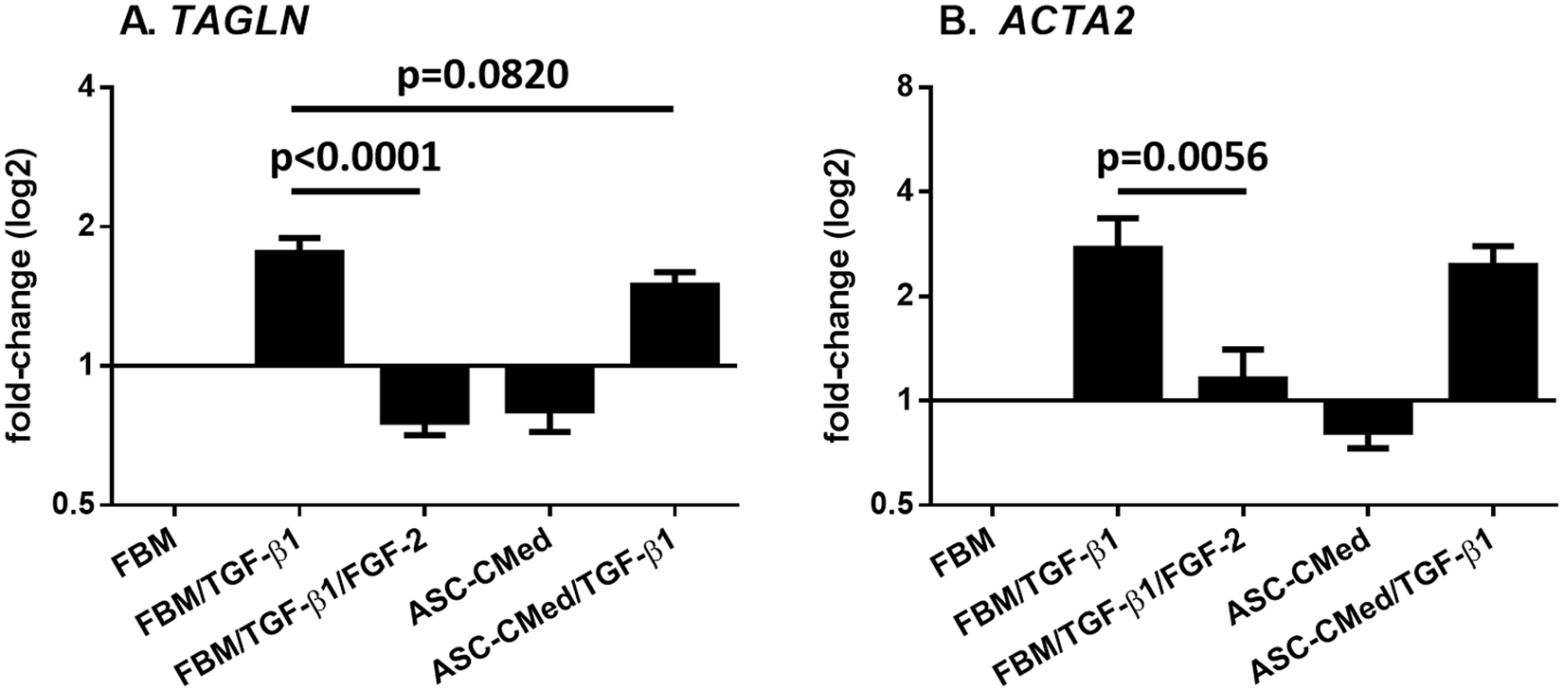
FGF-2, but not ASC-CMed, inhibits the gene expression of mesenchymal markers. (**A**) TAGLN, and (**B**) ACTA2, by RT-qPCR of NHCF-V after stimulation with TGFβ1 or co-stimulation with TGF-β1 and FGF-2, both in FBM and ASC-CMed, for five days. Data were analyzed by One-way ANOVA with Sidak’s multiple comparison test for the groups FBM/TGF-β1 vs. FBM/TGF-β1/FGF-2 and FBM/TGF-β1 vs. ASC-CMed/TGF-β1; p-values for the Sidak’s multiple comparison test are shown in the figure. Values represent mean ± SEM of 3 independent experiments in duplicate.

The expression of mesenchymal genes *TAGLN*, encoding SM22α, and *ACTA2*, encoding αSMA, was increased TGF-β1 stimulated fibroblasts, irrespective of ASC-CMed (Fig. 2). FGF-2 suppressed the expression of *TAGLN* (One-way ANOVA, p < 0.0001; Sidak’s multiple comparison test, p < 0.0001) and *ACTA2 in* cardiac fibroblasts stimulated with TGF-β1 (One-way ANOVA, p = 0.0007; Sidak’s multiple comparison test, p = 0.0056). The influence of ASC-CMed no more than tended to decrease *TAGLN* expression (One-way ANOVA, p < 0.0001; Sidak’s multiple comparison test, p = 0.0820) but not *ACTA2* expression.

**Figure 3:**
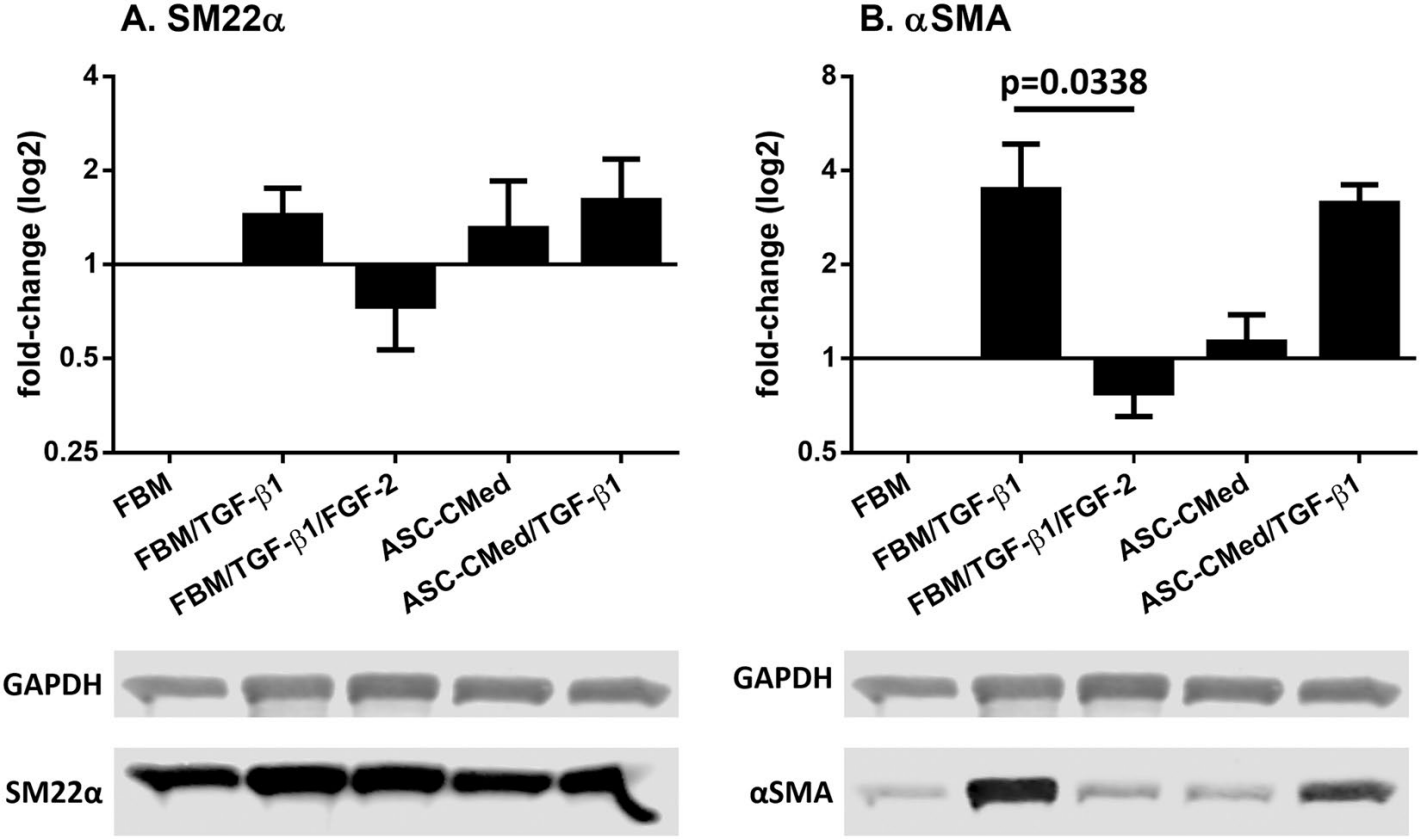
FGF-2, but not ASC-CMed, inhibits the protein expression of mesenchymal markers. (**A**) SM22α, and (**B**) αSMA, by immunoblotting of NHCF-V after stimulation with TGFβ1 or co-stimulation with TGF-β1 and FGF-2, both in FBM and ASC-CMed, for five days. Data were analyzed by One-way ANOVA with Sidak’s multiple comparison test for the groups FBM/TGF-β1 vs. FBM/TGF-β1/FGF-2 and FBM/TGF-β1 vs. ASC-CMed/TGF-β1; p-values for the Sidak’s multiple comparison test are shown in the figure. Values represent mean ± SEM of 3 independent experiments.

TGF-β1 upregulated the expression of αSMA in human cardiac fibroblasts which was blocked by FGF-2 (Fig. 3) (One-way ANOVA, p = 0.0413; Sidak’s multiple comparison test, p = 0.0338). ASC-CMed did not alter the upregulation of αSMA by TGF-β1 (Fig. 3). Under these culture conditions, cardiac fibroblasts had a basal expression of SM22α which increased 1.4-fold after TGF-β1 stimulation. Thus, although FGF-2 inhibits the production of αSMA, it could not reverse the expression of the already formed SM22α.

### FGF2, but not ASC-CMed, modulates extracellular matrix production in NHCF-V stimulated with TGF-β1

**Figure 4:**
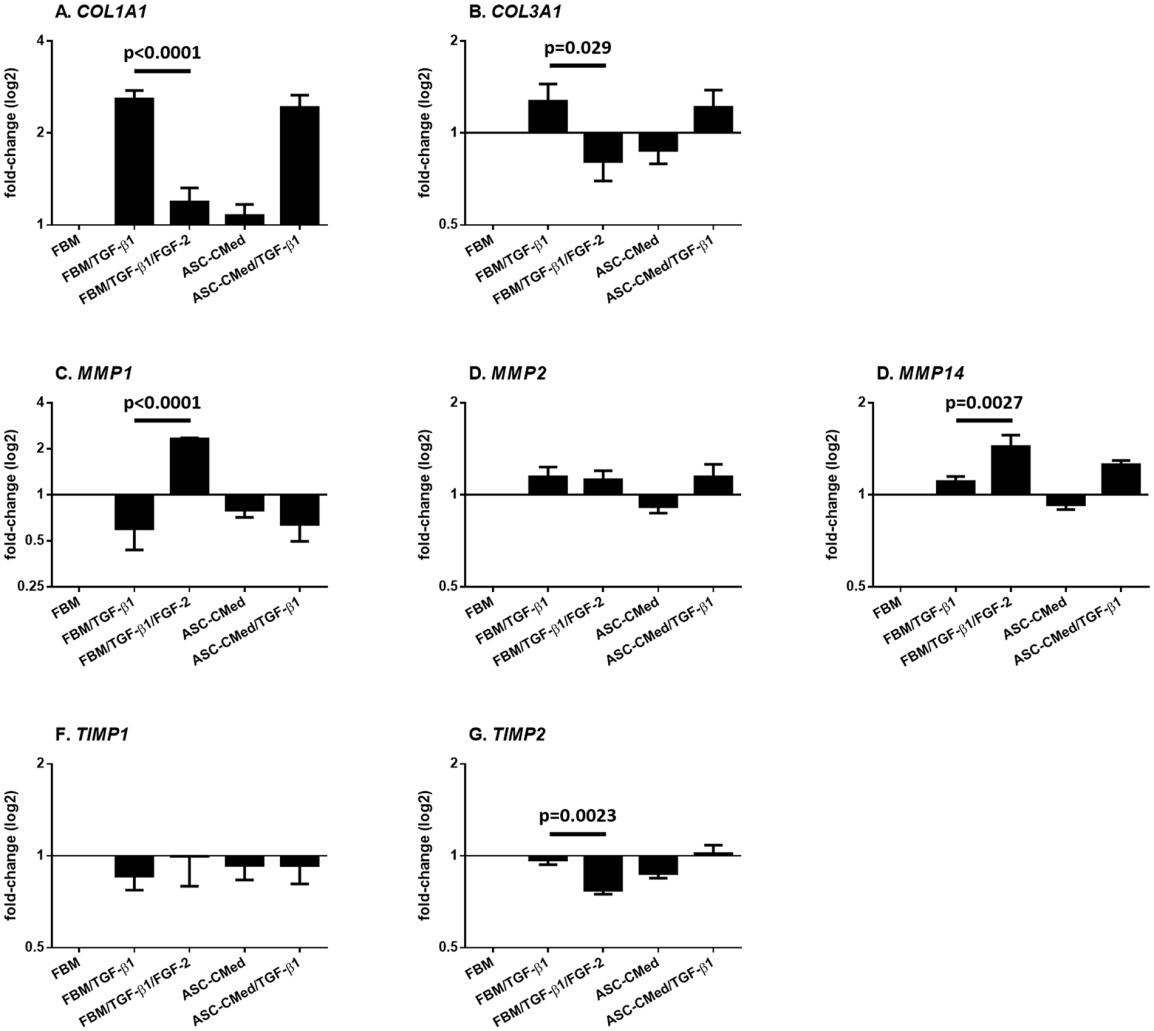
FGF2, but not ASC-CMed, modulates the expression of extracellular matrix-related genes. (**A**) *COL1A1*, (**B**) *COL3A1*, (**C**) *MMP1*, (**D**) *MMP2*, (**E**) *MMP14*, (**F**) *TIMP1*, and (**G**) *TIMP2* by RT-qPCR of NHCF-V after stimulation with TGF-β1 or co-stimulation with TGF-β1 and FGF-2, both in FBM and ASC-CMed, for five days. Data were analyzed by One-way ANOVA with Sidak’s multiple comparison test for the groups FBM/TGF-β1 vs. FBM/TGF-β1/FGF-2 and FBM/TGF-β1 vs. ASC-CMed/TGF-β1; p-values for the Sidak’s multiple comparison test are shown in the figure. Values represent mean ± SEM of 3 independent experiments in duplicate.

The gene expression of collagens, as well as of matrix metalloproteinases (MMPs) - enzymes responsible for ECM degradation - and the tissue inhibitors of metalloproteinases (TIMPs), was analyzed. The stimulation with TGF-β1 upregulated the transcription of both *COL1A1* and *COL3A1*, genes responsible for the protein synthesis of the alpha-1 chain of collagens I and III respectively (Fig. 4A,B). This increase was more pronounced for *COL1A1*. Treatment with FGF-2 in samples stimulated by TGF-β1 was responsible for a statistically significant downregulation in *COL1A1* (One-way ANOVA, p < 0.0001; Sidak’s multiple comparison test, p < 0.0001) and *COL3A1* (One-way ANOVA, p = 0.0572; Sidak’s multiple comparison test, p = 0.0290), demonstrating the strong inhibition of NHCF-V towards the myofibroblast phenotype.

The gene expression of MMPs and TIMPs did not change irrespective of treatment, except for FGF-2 (Fig. 4C–G). Expression of *MMP1*, encoding matrix metalloproteinase1 *i.e*. collagenase, was downregulated by TGF-β1. In contrast, FGF-2 upregulated *MMP1* expression compared to control groups and TGF-β1 stimulation (One-way ANOVA, p < 0.0001; Sidak’s multiple comparison test, p < 0.0001). Treatment with ASC-CMed did not affect the TGF-ß-induced downregulation of *MMP1* in NHCF-V. The expression of *MMP2* gene was not regulated by TGF-ß, FGF or ASC-CMed (co)stimulation of NHCF-V. Although TGF-β1 did not affect the expression of *MMP14*, FGF-2 upregulated its expression (One-way ANOVA, p < 0.0001; Sidak’s multiple comparison test, p = 0.0027). Treatment with ASC-CMed tended to increase the expression of *MMP14*.

Expression of TIMPs genes *TIMP1* and *TIMP2* which are regulators of MMP activity had differential expression. Expression of *TIMP1*, remained unchanged irrespective of TGF-ß1 or ASC-CMed treatment. Expression of *TIMP2* was decreased by FGF-2 (One-way ANOVA, p = 0.0005; Sidak’s multiple comparison test, p = 0.0023), while neither TGF-ß1 nor ASC-CMed affected its expression. Interestingly, TIMP2 is a co-factor of membrane-bound MMP14, which is e.g. responsible for activation of ECM-bound MMPs.

**Figure 5:**
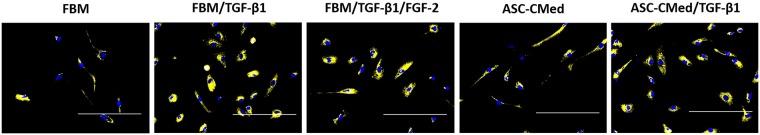
Pro-collagen production in cardiac fibroblasts. Immunofluorescence analysis of expression of pro-collagen in human cardiac fibroblasts undergoing myofibroblast differentiation for five days. Collagen I was upregulated upon TGF-β1 stimuli and neither ASC-CMed nor FGF-2 inhibited the process. Minor expression of collagen I was showed in cultured cells without TGF-β1 stimuli. Blue: DAPI; Yellow: collagen I. Scale reference: 200 μm.

Immunofluorescence microscopy of intracellular collagen I demonstrated an increase in the protein content for all the groups stimulated with TGF-β1 (Fig. 5). FGF-2 could not decrease the intracellular collagen I at the microscopical level, so that virtually all the TGF-β1 stimulated cells and ASC-CMed/TGF-β1 expressed the protein in their cytoplasm. Both groups without TGF-β1 stimulation showed a very limited expression of collagen I.

### FGF2 and ASC-CMed restore the TGF-ß1-inhibited proliferation of NHCF-V

**Figure 6:**
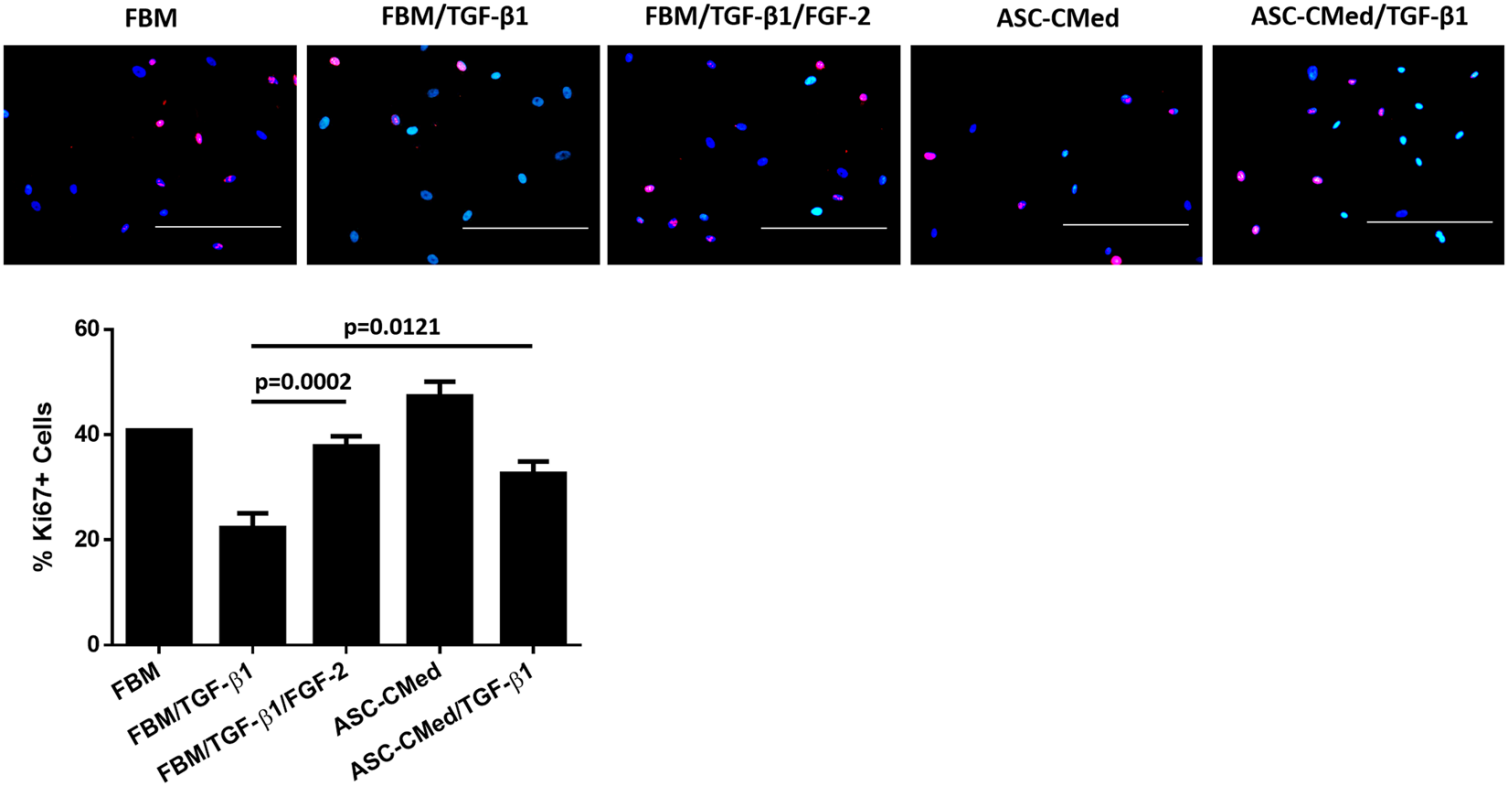
FGF2 and ASC-CMed restore the TGF-ß1-inhibited proliferation of NHCF-V. Immunofluorescence proliferation analysis of human cardiac fibroblasts undergoing myofibroblast differentiation for five days. TGF-β1 stimuli downregulated the proliferation of cardiac fibroblasts. ASC-CMed and FGF-2 upregulated the proliferation. Blue: DAPI; Red: Ki-67. Scale reference: 200 μm. Data were analyzed by One-way ANOVA with Sidak’s multiple comparison test for the groups FBM/TGF-β1 vs. FBM/TGF-β1/FGF-2 and FBM/TGF-β1 vs. ASC-CMed/TGF-β1; p-values for the Sidak’s multiple comparison test are shown in the figure. Values represent mean ± SEM of 3 independent experiments in quadruplicate.

NHCF-V proliferation was measured after five days of stimulation with TGF-β1. The pro-fibrotic stimulus led to a decrease in cell proliferation, as detected by Ki-67 staining. TGF-β1 suppressed the proliferation of human cardiac fibroblasts; control cells had 40.8% of proliferating cells compared to 22.3% in TGF-β1 stimulated cells (Fig. 6). Treatment with FGF-2, recovered the proliferation to 37.8% (One-way ANOVA, p < 0.0001; Sidak’s multiple comparison test, p = 0.0002) while the proliferation of fibroblasts cells stimulated with TGF-β1 was virtually rescued by ASC-CMed (32.6%) (One-way ANOVA, p < 0.0001; Sidak’s multiple comparison test, p = 0.0121). Treatment of nonstimulated cardiac fibroblasts with ASC-CMed increased proliferation to 47.3%.

### Wound healing is not affected by NHCF-V stimulation with TGF-β1

**Figure 7:**
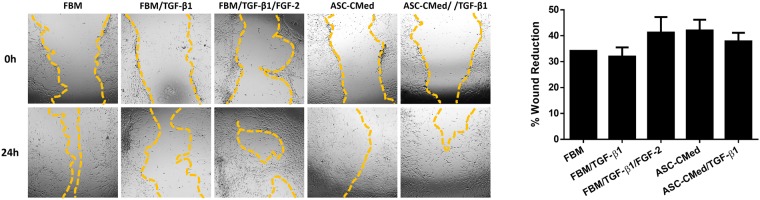
Wound healing is not affected by NHCF-V stimulation with TGF-β1. Wound healing assay (24 h) in NHCF-V under stimulation with TGF-β1 or co-stimulation with TGF-β1 and FGF-2, both in FBM and ASC-CMed. Original magnification: 4x. Values represent mean ± SEM of 3 independent experiments in triplicate.

Wound healing was examined with a scratch assay and assessed after 24 hours. The percentage of gap closure did not differ among all groups (One-way ANOVA, p = 0.3716). Still, the treatment with FGF-2 or ASC-CMed tended to increase wound healing potential (Fig. 7).

## Discussion

We demonstrated that FGF-2 - but not the secreted factors of adipose tissue-derived stromal cells – downmodulate TGF-ß1-induced fibroblast into myofibroblast differentiation. Our results show that FGF-2 reduced differentiation via the reduction of mesenchymal gene expression (*TAGLN* and *ACTA2*), and the reduction of αSMA expression subsequently. Concomitantly, we demonstrated that FGF-2 downregulated expression of collagens (*COL1A1* and *COL3A1*), upregulate expression of matrix metalloproteinases (*MMP1* and *MMP14*), and downregulated expression of inhibitor of metalloproteinase *TIMP2*. Finally, we showed that not only FGF-2 but also ASC-CMed restored the proliferation of TGF-β1-impaired fibroblasts.

TGF-β1 signaling is a pivotal mechanism of the activation of fibroblasts into myofibroblasts and, thus, fibrosis^6–10^. The ability of FGF-2 to antagonize TGF-β signaling, in turn, has been demonstrated in a series of studies^17,36–39^, each of these suggesting different mechanisms of interference on the TGF-β pathway. Among these suggested mechanisms are: the activation of ERK and JNK pathways^17^; the expression of *let-7* miRNA, TGFβ receptor suppressor^38,39^; the expression of miRNA-20a, another repressor of the TGFβ receptor complex^36^; and 4 the inhibition of the transcriptional regulator of muscle differentiation myf-5^40^.

We hypothesized that, because ASC are known to secrete pro-regenerative growth factors such as FGF-2, treatment of human cardiac fibroblasts with ASC-CMed would abrogate TGF-β1-induced differentiation into myofibroblasts. Both gene and protein expression of mesenchymal and extracellular matrix-related markers showed that, differently than expected, ASC-CMed did not exert a noticeable effect on TGF-β1-induced myofibroblast activation. We did find, however, that ASC-CMed restored fibroblast proliferation impaired by TGF-β1 stimulation. This finding might point to potential differences between the FGF-2 thresholds required for restoring proliferation and blocking differentiation. However, another possibility is that ASC produce other growth factors that also impact proliferation but are not involved in the differentiation process. In this regard, it has been previously shown that epidermal growth factor (EGF) from ASC was capable to enhance the proliferation of skin fibroblasts *in vitro*^41^.

To our knowledge, this is the first report to investigate the effects of conditioned medium from mesenchymal stromal cells - here ASC - in the TGF-β1-induced differentiation of cardiac fibroblasts into myofibroblasts. Several other studies, however, have investigated the effects of MSC secretome in the spontaneous differentiation of cardiac fibroblasts^42–46^. Thus, although our findings oppose to many of the results described in these studies, there are important differences - which deserve attention - between published data and our current work (Table 1).

**Table 1:** Overview of the studies investigating the effects of MSC conditioned medium in cardiac fibroblasts.

Study	Fibroblast type	Fibroblast culture medium	MSC conditioned medium	Differentiation induction	Findings
Ohnishi *et al*.^42^	primary SD rat atrial fibroblast	αMEM (10% FBS)	αMEM (10% FBS)-based CM from Lewis rat BM-MSC cultured for 48 h	spontaneous	= proliferation = *COL1A1* and *COL3A1* gene expression
Mias *et al*.^43^	primary Lewis rat cardiac^(NS)^ fibroblast	DMEM/F12 (10% FBS)	MEM (10% FBS)-based CM from Lewis rat BM-MSC cultured for 24 h*	spontaneous	↓ αSMA protein expression ↓ collagens I and III deposition ↑ MMP2 and MMP9 activity ↓ *TIMP2* gene expression
Wang *et al*.^44^	primary SD rat ventricular fibroblast**	DMEM (10% FBS)	transwell co-culture with SD rat BM-MSC in DMEM (10% FBS)	spontaneous	↓ MMP2 protein expression = MMP9 protein expression ↓ MMP2 activity = MMP9 activity ↑ TIMP1 protein expression
Mao *et al*.^45^	primary SD rat ventricular fibroblast	DMEM (10% FBS)	DMEM (10% FBS)-based CM from SD rat BM-MSC cultured for 72 h and concentrated***	spontaneous	↓*ACTA2* gene expression ↓ *COL1A1* and *COL3A1* gene expression ↑ *MMP2* and *MMP9* gene expression ↓ *TIMP1* and *TIMP2* gene expression
Li *et al*.^46^	primary SD rat ventricular fibroblast	DMEM (10% FBS)	gap mode co-culture with human ASC in MSC growth medium^(NS)^	spontaneous	↓ αSMA expression
Liguori *et al*. (current paper)	primary human ventricular fibroblast	FGM (10% FBS)	DMEM (10% FBS)-based CM from human ASC cultured for 24 h, concentrated 30X, and resuspended in FGM (2% FBS)	5 ng/mL of TGF-β1	= *TAGLN* and *ACTA2* gene expression = SM22α and αSMA protein expression = *COL1A1* and *COL3A1* gene expression = *MMP1*, *MMP2* and *MMP14* gene expression = *TIMP1* and *TIMP2* gene expression ↑ proliferation = wound healing potential

MSC: mesenchymal stromal cells; SD: Sprague-Dawley; αMEM: α-Minimal Essential Medium; FBS: fetal bovine serum; CM: conditioned medium; BM-MSC: bone marrow mesenchymal stromal cells; DMEM: Dulbecco’s Modified Eagle Media; MEM: Minimal Essential Medium; ASC: adipose tissue-derived stromal cells; FGM: Fibroblast Growth Medium;

NS: not specified.

*Used the same medium, not conditioned, as control.

**Fibroblasts pre-conditioned under hypoxia.

***Concentration ratio and resuspension method (if any) not described.

Three out of the five studies reported anti-fibrotic effects of MSC conditioned-medium on cardiac fibroblasts, including a reduction in expression of αSMA, collagens and TIMPs and increased MMPs expression and/or activity^43,45,46^. These studies were all performed with rat - not human - cardiac fibroblasts and none of these included a control group treated with Fibroblast Growth Medium (FGM), a medium capable to inhibit the spontaneous differentiation of fibroblasts, as we did. Thus, in this respect our results are not comparable to these studies for several reasons, the main being that the induction of cardiac fibroblast differentiation with TGF-β1 is a much more effective and powerful mechanism than the spontaneous differentiation, being consequently much more difficult to be blocked or reversed. Furthermore, both other studies which investigated the effects of MSC conditioned-medium on the spontaneous differentiation of cardiac fibroblasts reported either no differences^42^ or even a trend towards the pro-fibrotic phenotype, with reduced MMP2 and MMP9 protein expression and activity and increased TIMP1 protein expression^44^. An important observation is that the concentration of FGF-2 in the ASC-CMed could be too low to counteract TGF-β1 action, while it is enough to block spontaneous differentiation. Previous studies demonstrated that the concentration of FGF-2 in ASC-CMed was less than 150 pg/mL^33,34^, much lower than the FGF-2 concentration necessary to block the TGF-β1-induced differentiation in this study. Another point which deserves attention is the variety of factors secreted by ASC which could interfere with FGF-2 activity. Among the factors that might be involved in the FGF-2 blockage of TGF-β1 signaling are inflammatory factors, as INFα, TNFα, and IL-1β, which block the FGF receptor and, thus, do not allow FGF-2 to inhibit TGF-β1 action^39^. In fact, studies demonstrated that ASC may release TNFα and IL-1β, besides many other interleukins^33,47,48^. These factors, together, could abrogate the effect of FGF-2 in the low doses it is found in ASC-CMed. Still, it is essential to highlight the fact that ASC also produce TGF-β, as demonstrated by several studies^33,34,49,50^. In this regard, Rehman *et al*. showed that ASC produce around ten times more TGF-β than FGF-2^34^. The conjoint action of all these factors, which are contained in the ASC secretome, could explain, in part, the insufficiency of ASC-CMed to block the fibrotic stimule.

Still, in our view, the partial recapitulation *in vitro* of the fibroblast-related component of cardiac fibrosis, with the use of primary human cardiac fibroblast cultured in FGM and induced with TGF-β1 is a suitable *in vitro* model of the natural conditions in the fibrotic heart. Thus, considering the controversial findings between the studies using the spontaneous differentiation in addition to the results of the present study, we consider that in TGF-β-driven cardiac fibrosis ASC-CMed may not suffice as a sole therapeutic modality to block or reverse ongoing fibrosis. FGF-2, however, corroborating with the findings of two previous studies^21,22^, did block TGF-β1-induced pro-fibrotic conversion of cardiac human fibroblasts.

The present study supports the anti-fibrotic effects of FGF-2 through the blockage of NHCF-V differentiation into myofibroblasts, as demonstrated by the modulation of gene and/or protein expression in a series of mesenchymal and extracellular matrix-related markers. ASC-CMed, however, could not demonstrate the same effects found with FGF-2.

## Methods

### Experimental groups

Ventricular normal human cardiac fibroblasts (NHCF-V) were allocated into 5 groups with different induction/FGF-2/ASC-CMed combinations, as described in Table 2.

**Table 2:** Experimental Groups.

Group	Description
FBM	NHCF-V culture with FGM2, without added factors.
FBM/TGF-β1	NHCF-V culture with FGM2 added with TGF-β1.
FBM/TGF-β1/FGF-2	NHCF-V culture with FGM2 added with TGF-β1 and FGF-2
ASC-CMed	NHCF-V culture with ASC conditioned media, without added factors.
ASC-CMed/TGF-β1	NHCF-V culture with ASC conditioned media added with TGF-β1

FBM: Fibroblast media; FGM2: Fibroblast growth media with 2% of fetal bovine serum; NHCF-V: Ventricular normal human cardiac fibroblasts; FGF-2: Fibroblast growth factor 2; TGF-β1: transforming growth factor-beta 1; ASC-CMed: Conditioned Medium derived from Adipose tissue-derived stromal cells.

### Cell sources, cell culture, and conditioned medium

Ventricular normal human cardiac fibroblasts were purchased from Lonza (NHCF-V; #CC-2904, Lonza, Basel, Switzerland) and cultured with Fibroblast Growth Medium-3 with 10% of fetal bovine serum (FBS) (FGM3; #CC-4526, Lonza, Basel, Switzerland) added with 500 ng/mL Amphotericin B (#15290018, Gibco Invitrogen, Carlsbad, USA) at 37 °C in a humidified incubator with 5% CO_2_. The medium was refreshed every 3 days. Cells were passed at a ratio of 1:3 after reaching confluency. NHCF-V were used between passages 3–5.

At day 0 of the experiment, NHCF-V were seeded at a 10,000 cells/cm^2^ density. After 24 hours, NHCF-V were allocated into the 5 distinct groups and cultured for 5 days. Fibroblast growth factor 2 (FGF-2; #100-18C, PeproTech, Inc., Rocky Hill, N.J.) and human transforming growth factor-beta 1 (TGF-β1; #100-21, PeproTech, New Jersey, USA) were used at a concentration of 10 ng/mL and 5 ng/mL, respectively, in all experiments.

Human ASC were isolated as described previously^51^. Briefly, human abdominal fat was obtained by liposuction, washed with phosphate-buffered saline (PBS) and digested enzymatically with 0.1% collagenase A (#11088793001, Roche Diagnostic, Mannheim, Germany) in PBS with 1% bovine serum albumin (BSA; #A9647, Sigma-Aldrich, Boston, USA). The tissue was shaken constantly at 37 °C for 2 h. After this, the digested tissue was mixed with 1% PBS/BSA, filtered, centrifuged and the cell pellet was resuspended in Dulbecco’s Modified Eagle’s Medium (DMEM; #12-604F, Lonza, Basel, Switzerland) with 10% fetal bovine serum (FBS; #F0804, Sigma-Aldrich, Missouri, United States), 1% penicillin/streptomycin (#15140122, Gibco Invitrogen, Carlsbad, USA) and 1% L-glutamine (#17-605E, Lonza Biowhittaker, Verviers, Belgium). Cells were cultured at 37 °C in a humidified incubator with 5% CO_2_. The medium was refreshed every 2 days. Cells were passed at a ratio of 1:3 after reached confluency.

ASC conditioned medium (ASC-CMed) was obtained from confluent cultures of ASC between passages 3 and 6 from at least 3 different donors. For ASC-CMed, cells were cultured in DMEM without serum. Cells were kept at 37 °C with a minimum relative humidity of 95% and an atmosphere of 5% CO_2_ in the air. The conditioned medium was harvested after 24 hours, passed through a 0.22 µm filter and stored t −20 °C until use. Before the experiment, the conditioned medium was concentrated 30 times using Amicon® Ultra 15 mL filters (UFC900324, Merck, Darmstadt, Germany) and resuspended to the initial volume using Fibroblast Growth Medium-2 (FGM2; #CC-3132, Lonza, Basel, Switzerland).

### Immunofluorescence, gene expression, and immunoblotting

#### Immunofluorescence

NHCF-V were cultured in 48 well tissue culture plates. After 5 and 21 days of induction, cells were fixed at room temperature with 2% paraformaldehyde (PFA) for 30 minutes. Cells were permeabilized with 1% Triton-X100 in PBS for 15 minutes at room temperature and blocked with 5% donkey serum in PBS and 1% BSA for another 15 minutes at room temperature. Subsequently, cells were incubated with primary antibodies diluted in 5% donkey serum in PBS for 2 hours at room temperature. The following primary antibodies were used: mouse anti-αSMA (1:400; #ab5694, Abcam, Cambridge, UK), rabbit anti-collagen I (1:200, #ab34710, Abcam, Cambridge, UK), rabbit anti-collagen III (1:200, #ab34710, Abcam, Cambridge, UK) and rabbit anti Ki-67 (1:400; #ab15580, Abcam, Cambridge, UK). Controls were incubated with 5% donkey serum in PBS. Next, cells were washed with 0.05% Tween-20 in PBS and incubated with secondary antibodies in 5% donkey serum in PBS with 4′,6-diamidino-2-phenylindole (DAPI; 1:5000; #D9542-5MG, Sigma-Aldrich, Missouri, United States) and Alexa Fluor® 488 phalloidin (1:400; #A12379, Life Technologies, Carlsbad, United States) for 1 hour at room temperature. The following secondary antibodies were used: donkey anti-rabbit IgG (H + L) Alexa Fluor® 594 (1:400; #A-21207, Life Technologies, Carlsbad, United States) and donkey anti-mouse IgG (H + L) Alexa Fluor® 647 (1:400; #A-31571, Life Technologies, Carlsbad, United States). Finally, cells were washed 3 times with PBS and the plates were imaged with Evos FL System (Thermo Fisher Scientific, Waltham, United States) using Texas Red (TXR), Cy5, DAPI and Green Fluorescent Protein (GFP) channels with 20x magnification.

#### Gene expression analysis

NHCF-V were cultured in 25 cm^2^ flasks. After 5 days of induction, RNA isolation was performed using TRIzol reagent (#15596018, Invitrogen Corp, Carlsbad, United States) according to the manufacturer’s protocol. RNA concentration and purity were determined using NanoDrop technology (Thermo Scientific, Hemel Hempstead, United Kingdom). Between 300 ng and 500 ng of total RNA was used for cDNA synthesis, which was performed using RevertAid^TM^ First Strand cDNA Synthesis Kit (Thermo Fisher Scientific, Waltham, United States) according to the manufacturer’s protocol. The cDNA-equivalent of 5 ng total RNA was used per single qPCR reaction. PCR was performed using SYBR Green (Bio-Rad, Hercules, United States) with the ViiA7 Real-Time PCR system (Applied Biosystems, Foster City, United States). Each analysis was done in duplicate for each one of the independent experiments. The primers used are listed in the Supplementary Table S1. Data were analyzed using ViiA7 software (Applied Biosystems, Foster City, United States) and normalized with the ∆Ct method, using the geometrical mean of 18S ribosomal RNA (*18S RNA*) cycle threshold (C_T_) values. The fold-change in gene expression versus the no treatment control group (ECM) was calculated using the ∆∆C_T_ method.

#### Immunoblotting analysis

NHCF-V were cultured in 25 cm^2^ flasks. After 5 days of induction, cells were rinsed with cold PBS and lysed in 100 µL of cold lysis buffer (RIPA; #89900, Thermo Fisher Scientific, Waltham, United States) containing 1% protease inhibitor cocktail (PIC; #P8340, Sigma Aldrich, St. Louis, United States) and 1% Halt™ phosphatase inhibitor cocktail (#78420, Thermo Fisher Scientific, Waltham, United States). The lysed cells were collected in 2 mL microcentrifuge tubes and the contents homogenized by sonication at 30 W for 30 seconds and centrifuged at 7,500 g at 4 °C for 5 minutes. The supernatant was collected for the protein concentration determination using the Bio-Rad DC protein assay (#5000112; Bio-Rad, Hercules, United States) according to the manufacturer’s protocol. Gels (12%) were loaded with 4–10 μg of protein. After electrophoresis, gels were blotted onto nitrocellulose membranes (#170-4270; Bio-Rad, Hercules, United States). Blots were blocked with Odyssey® Blocking Buffer (#927-40000, LI-COR, Lincoln, USA) in a dilution of 1:1 with PBS overnight at 4 °C. Afterward, blots were incubated with the primary antibodies overnight. The following primary antibodies were used: rabbit anti-SM22α (1:1000; #ab14106, Abcam, Cambridge, UK), mouse anti-αSMA (1:1000; #ab5694, Abcam, Cambridge, UK) and rabbit anti-GAPDH (1:1000; #ab9485, Abcam, Cambridge, UK). Then, the membranes were washed during 30 minutes with Tris-buffered saline with 0.1% Tween-20 (TBST) and incubated for 1 hour with the Odyssey® secondary antibodies goat anti-rabbit IRDye 680LT (1:10000; #926-68021, LI-COR, Lincoln, USA) and goat anti-mouse IRDye 800CW (1:10,000; #926-32210, LI-COR, Lincoln, USA). Non-bound secondary antibodies were removed by washing with TBST for 30 minutes. Then, blots were washed for 5 minutes with Tris-buffered saline (TBS) and scanned with Odyssey® Infrared Imaging System (LI-COR, Lincoln, USA).

### Wound healing assay

NHCF-V were seeded in 48-well tissue culture plates at a density of 15,000 cells/cm^2^ and cultured in FGM3, 37 °C, and 5% CO_2_ until 70% confluence was reached. Them, cells were divided into 5 groups and stimulated for 5 days, as prescribed previously. After this period, the confluent monolayers were scored with a 10 µl sterile pipette tip to leave a scratch of approximately 0.4–0.5 mm in width. Culture medium was, then, immediately removed and cells were carefully washed with PBS - to remove any dislodged cells - and the medium was replaced with fresh FGM2. Pictures were taken immediately after the scratch and, again, after 24 hours. The percentage of wound reduction was evaluated in ImageJ.

### Statistical analysis

All data were obtained from at least three independent experiments, performed in duplicate or triplicate. Data are presented as mean ± standard error of the mean (SEM). Graphs and statistical analysis were done using GraphPad Prism (Version 6.01; GraphPad Software, Inc., La Jolla, United States). Differences among multiple groups were analyzed by One-way ANOVA with Sidak’s multiple comparison test for the following groups: FGM2/TGF-β1 vs. FGM2/TGF-β1/FGF-2 and FGM2/TGF-β1 vs. ASC-CMed/TGF-β1.

## Electronic supplementary material

Supplementary Material

## Data Availability

The datasets generated during and/or analyzed during the current study are available from the corresponding author on reasonable request.
