# Supplementary material for: Fibroblast growth factor-2, but not the adipose tissue-derived stromal cells secretome, inhibits TGF-β1-induced differentiation of human cardiac fibroblasts into myofibroblasts
Source: Sci Rep. 2018 Nov 9;8:16633. doi: 10.1038/s41598-018-34747-3 (PMC6226511; doi:10.1038/s41598-018-34747-3)
Supplement: Supplementary file 1 — Supplementary Material [file 41598_2018_34747_MOESM1_ESM.pdf]

Fibroblast growth factor-2, but not the adipose tissue-derived stromal cells secretome, inhibits TGF-β1-induced differentiation of human cardiac fibroblasts into myofibroblasts

Tacia Tavares Aquinas Liguori[1,2+], Gabriel Romero Liguori[1,2+], Luiz Felipe Pinho Moreira[1], Martin Conrad Harmsen[2]*

1. Laboratório de Cirurgia Cardiovascular e Fisiopatologia da Circulação (LIM-11), Instituto do Coração (InCor), Hospital das Clinicas HCFMUSP, Faculdade de Medicina, Universidade de Sao Paulo, Sao Paulo, SP, BR

2. University of Groningen, University Medical Center Groningen, Department of Pathology and Medical Biology, Groningen the Netherlands.

[+] These authors equally contributed to the manuscript.

***Corresponding author:**

Prof.dr. M.C. Harmsen

University of Groningen, University Medical Center Groningen, Dept. Pathology and Medical Biology, Hanzeplein 1 - EA11, 9713 GZ Groningen, the Netherlands.

e-mail: m.c.harmsen@umcg.nl

telephone: +31503614776/FAX: +31503619911

**Supplementary Table S1. Primer Sequences of Genes**			
	**Gene**	**Forward Primer Sequence (5' – 3')**	**Reverse Primer Sequence (5' – 3')**
**Reference Gene**	***GAPDH***	CTTGCCATCCTTCTCAAAGT	GCCCAATACGACCAAATCC
**Mesenchymal Markers**	***TAGLN***	CTGAGGACTATGGGGTCATC	TAGTGCCCATCATTCTTGGT
	ACTA	CTGTTCCAGCCATCCTTCAT	TCATGATGCTGTTGTAGGTGGT
**ECM Markers**	***COL1A1***	GGGATTCCCTGGACCTAAAG	GGAACACCTCGCTCTCCA
	COL3A1	CTGGACCCCAGGGTCTTC	CATCTGATCCAGGGTTTCCA
	TIMP1	CCAGCGTTATGAGATCAAGA	AGTATCCGCAGACACTCTCC
	TIMP2	GAAGAGCCTGAACCACAGGT	CGGGGAGGAGATGTAGCAC
	MMP1	GCTAACCTTTGATGCTATAACTACGA	TTTGTGCGCATGTAGAATCTG
	MMP2	GTTCCCCTTCTTGTTCAATG	CTTGCCATCCTTCTCAAAGT
	MMP14	GGGTGAGGAATAACCAAGTG	CTTCCTCTCGTAGGCAGTGT
